# Targeting Cyclin-Dependent Kinases in Synovial Sarcoma: Palbociclib as a Potential Treatment for Synovial Sarcoma Patients

**DOI:** 10.1245/s10434-016-5341-x

**Published:** 2016-06-22

**Authors:** Myrella Vlenterie, Melissa H. S. Hillebrandt-Roeffen, Esther W. M. Schaars, Uta E. Flucke, Emmy D. G. Fleuren, Anna C. Navis, William P. J. Leenders, Yvonne M. H. Versleijen-Jonkers, Winette T. A. van der Graaf

**Affiliations:** 1 Department of Medical Oncology, Radboud University Medical Center, Nijmegen, The Netherlands; 2 Department of Pathology, Radboud University Medical Center, Nijmegen, The Netherlands; 3 The Institute of Cancer Research London and the Royal Marsden NHS Foundation Trust, London, UK

## Abstract

**Background:**

In synovial sarcomas alterations in the cyclin D1-CDK4/6-Rb axis have been described. Also, β-catenin, a cyclin D1 regulator, is often overexpressed. Additionally, studies have shown that the t(X;18) translocation influences tumor behavior partly through cyclin D1 activation. We investigated how alterations in the cyclin D1-CDK4/6-Rb axis impact prognosis and studied effects of targeting this axis with the CDK4/6 inhibitor palbociclib.

**Methods:**

Synovial sarcoma samples (*n* = 43) were immunohistochemically stained for β-catenin, cyclin D1, p16, p21, p27, Rb, and phospho-Rb. Fluorescent in situ hybridization (FISH) was performed to detect *CCND1* amplification or translocation. In 4 synovial sarcoma cell lines sensitivity to palbociclib was investigated using cell viability assays, and effects on the sensitive cell lines were evaluated on protein level and by cell cycle arrest.

**Results:**

Expression of nuclear phospho-Rb and nuclear β-catenin in the patient samples was associated with poor survival. FISH showed a sporadic translocation of *CCND1* in a subset of tumors. An 8-fold *CCND1* amplification was found in 1 cell line, but not in the patient samples investigated. Palbociclib effectively inhibited Rb-phosphorylation in 3 cell lines, resulting in an induction of a G1 arrest and proliferation block.

**Conclusions:**

In this series nuclear phospho-Rb and nuclear β-catenin expression were negative prognostic factors. In vitro data suggest that palbociclib may be a potential treatment for a subset of synovial sarcoma patients. Whether this effect can be enhanced by combination treatment deserves further preclinical investigations.

**Electronic supplementary material:**

The online version of this article (doi:10.1245/s10434-016-5341-x) contains supplementary material, which is available to authorized users.

Synovial sarcoma (SyS) is a subtype of soft tissue sarcomas (STS), characterized by a chromosomal translocation t(X;18). Localized disease is treated with surgery, occasionally with additional radiotherapy or chemotherapy. Palliative chemotherapy can be given once patients have advanced disease, with poor survival rates. Although the field of targeted treatment for carcinomas is developing rapidly, trials with targeted therapies for rare cancers such as sarcoma are scarce, and pazopanib is currently the only registered targeted treatment for non-gastrointestinal stromal tumor (GIST) STS, including SyS.^1^ To improve prognosis for this group of patients, knowledge on actionable targets in this sarcoma subtype is desperately needed. Among others, cyclin D1 has been suggested as a potential therapeutic target. Cyclin D1 is a cell cycle regulator essential for progression from G1 to S phase. Deregulated expression via mutations, gene rearrangements, or amplification of *CCND1* has been reported in various cancer types.^2^–^4^ Also alterations of other proteins involved in the cyclin D1-CDK4/6-Rb axis (e.g., p16, retinoblastoma (Rb) protein, and p21) may lead to uncontrolled progression through the cell cycle.^5^,^6^ Even though the exact working mechanism of the SyS translocation is still unknown, it has been shown that the SS18-SSX fusion gene is associated with cyclin D1 expression in SyS cells. Downregulation of the fusion gene expression by siRNA drastically decreased cyclin D1 levels, resulting in reduced cell proliferation.^7^–^9^ To further investigate the axis through which cyclin D1 is affected, several mechanisms have been postulated.

Cai et al. demonstrated that siRNA-mediated downregulation of the SS18-SSX2 gene product in SyS cell lines resulted in a decreased expression of (phospho)-ERK1/2 and cyclin D1, suggesting a direct link between this translocation and ERK1/2 and cyclinD1 activation.^10^ This was supported by a study showing that inhibition of the MEK/ERK-pathway with sorafenib led to downregulation of the expression of cyclin D1 and Rb protein with consecutive cell cycle arrest.^11^ It has also been suggested that the translocation associates with deregulated cyclin D1 activity via the Wnt/β-catenin-pathway. Indeed, this “cellular dedifferentiation” pathway is constitutively active in a SYT-SSX2 transgenic mouse model, with corresponding cyclin D1 expression, and inhibition of Wnt signaling through functional knock-out of the β-catenin gene reduced tumor formation.^12^,^13^ In line with this, blocking β-catenin with small molecule inhibitors in SyS cell lines resulted in cyclin D1 downregulation.^14^ Moreover, cyclin D1 activity could also be regulated by the PI3K/Akt-pathway. In vitro, PI3K inhibition resulted in decreased cell proliferation, which was linked to reduced cyclin D1 and increased levels of p27.^15^ Others have shown that cyclin D1-positive and nuclear β-catenin negative SyS were positive for phospho-Akt.^16^ It has also been suggested that the SSX2 gene product stimulates miR-17 expression, which subsequently inhibits p21.^17^ Additionally, heterozygous loss of p16 has been reported in SyS tumors, and recently we reported the occurrence of a *CCND1* mutation with additional nuclear overexpression of cyclin D1 in a patient sample.^18^–^20^

All these studies suggest that the cyclin D1-CDK4/6-Rb axis integrates inputs from the Wnt/β-catenin, PI3K/AKT, and ERK-pathways in SyS making the cyclin D1-CDK4/6-Rb axis an interesting target for treatment of SyS patients. However, so far no published data is available on the specific exploration of inhibiting this target in SyS. One such potential active drug could be palbociclib. Palbociclib (PD-0332991) is a highly selective drug, inhibiting the CDK4/6-cyclin D1 kinase activity while showing little or no activity to other enzymes. It was proven to reduce Rb phosphorylation at the 2 sites that are specifically phosphorylated by CDK4 and CDK6. Significant antitumor activity was detected only at doses where there was a sustained and significant reduction of these sites on Rb.^21^ Palbociclib has recently been FDA approved for the treatment of postmenopausal women with estrogen receptor (ER)-positive, human epidermal growth factor receptor 2 (HER2)-negative advanced breast cancer as initial therapy in metastatic disease in combination with letrozole. In sarcoma, the only phase 2 studies have been performed in liposarcoma.^22^ In SyS, no study on palbociclib has been done yet. Therefore, we here evaluated the cyclin D1-CDK4/6-Rb axis and tested palbociclib as a possible treatment for SyS patients.

## Materials and Methods

### Patient Characteristics

Tumor samples from 43 patients, diagnosed with SyS between 1988 and 2015, were retrieved from the files of the department of Pathology of the Radboud University Medical Centre (Radboud UMC). Patient follow-up data was retrieved from the clinical records. The study was performed in accordance with the Code of Conduct of the Federation of Medical Scientific Societies in the Netherlands.

### Immunohistochemistry

**Fig. 1:**
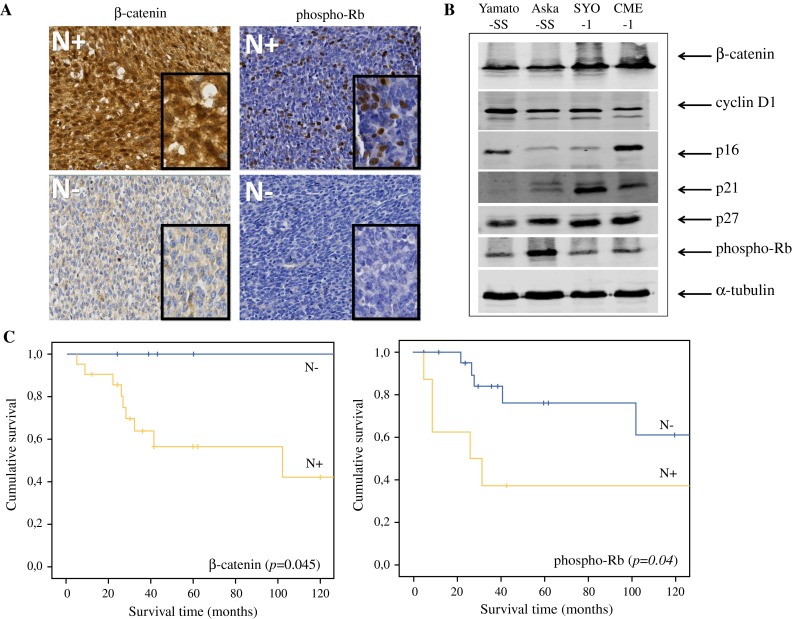
Proteins involved in the cyclin D1-CDK4/6-Rb axis. **A** Both nuclear (N) staining of β-catenin and nuclear (N) staining of phospho-Rb was scored as negative or positive. The cutoff was set at staining in at least 20 % of the cells. All images are taken at ×200 magnification, insets are at ×400 magnification. **B** Levels of the proteins involved in the cyclin D1-CDK4/6-Rb axis on western blot. Different expression levels can be seen between the 4 synovial sarcoma cell lines. **C** Kaplan–Meier survival curves for β-catenin and phospho-Rb expression levels, which resulted in an overall survival difference

Immunohistochemistry for cyclin D1, p16, p21, p27, β-catenin, Rb, and phospho-Rb was performed on 4-µm thick formalin-fixed paraffin-embedded (FFPE) tissue microarray (TMA) sections using standard procedures. The specifications for each antibody are listed in Supplemental Table 1. After incubation with primary antibody and washing, antibodies were visualized using PowerVision poly-HRP-anti-Ms/Rb/Rt (ImmunoLogic), visualized using bright 3,3′-diaminobenzidine (DAB, ImmunoLogic) and counterstained with hematoxylin. Immunostaining was scored by 3 individual researchers, and consensus was reached (scoring systems are explained below in Fig. 1 and Supplemental Fig. 1). Only results from primary tumors were included in the analysis, in order to obtain a representative view on survival. A Kaplan–Meier analysis was performed with a log-rank test to calculate survival differences, which were considered to be significant at *p* < 0.05.

### FISH

For the detection of gene rearrangements, fluorescent in situ hybridization (FISH) was performed on 2-µm thick FFPE TMA sections that were pretreated in sodium citrate buffer (pH6) by heating in a microwave oven for 10 min, followed by a digestion step with pepsin (200 U/ml) for 15 min at 37 °C. The *CCND1* DNA split probe (Y5414, DAKO) was applied to the sections and heat denatured for 5 min at 82 °C and hybridized at 45 °C overnight. Sections were mounted in Vectashield mounting medium with DAPI (Vector Laboratories). Fifty cells were counted, and >10 % fusion breakage was considered a translocation. Amplifications were considered when >6 *CCND1* signals were detected in >10 % of the cells.

### Cell Culture

The 2 patient-derived SyS cell lines harboring the SSX1 translocation (Yamato-SS and Aska-SS) were generously provided by K. Itoh. The 2 patient-derived SyS cell lines harboring the SSX2 translocation (SYO-1 and CME-1) were generously provided by A. Kawai and C. Lanzi, respectively. Yamato-SS and Aska-SS were cultured in DMEM 4.5 g/l glucose (Lonza), 1 mM sodium pyruvate (PAA) medium, SYO-1 in DMEM 4.5 g/l glucose, and CME-1 in RPMI-1640 medium (Lonza). Media were supplemented with 10 % fetal bovine serum (FBS, Gibco) and 1 % penicillin/streptomycin (Lonza). All cells were maintained at 37 °C in a humidified atmosphere of 5 % CO_2_/95 % air.

### MTT Assay

Cell viability (defined as metabolic cell activity) was assessed by MTT assay. Cells were seeded in triplicate in 96-well plates overnight for attachment and then incubated for 2 times the doubling time of the cell line, with palbociclib (PD-0332991, Pfizer) (0–10,000 nM). Next, MTT (Sigma-Aldrich) was added to each well and cells were incubated for 3.5 h. Afterward, the formazan crystals were dissolved in MTT solvent (isopropanol with 0.1 % Nonidet P-40 and 4 mM fuming hydrochloric acid) and absorbance was measured at 560 nm using an ELISA reader. IC_50_ values were calculated using GraphPad Prism Version 5.03 software.

### Cell Cycle Assay

SyS cells were dissociated with trypsin and fixated in 70 % ethanol after treatment with different concentrations of palbociclib at different time points. Propidium iodide solution (Sigma-Aldrich, 50 μg/ml in PBS) was added to the fixated cells to achieve DNA staining, and cells were treated with RNAse A (Qiagen, 100 μg/ml). All samples were measured using flow cytometry (CyAn ADP Analyzer, Beckman Coulter) and analyzed by FlowJo software (version 10). Hereby viable single cells were selected by gating.

### Western Blot

After treatment of cells with different concentrations of palbociclib, cells were trypsin-harvested and proteins extracted with RIPA buffer (Cell Signaling Technology), supplemented with 1 mM PMSF were loaded (40 μg of cell extract, determined using the BCA protein assay kit, Thermo Fisher Scientific) and separated by SDS-PAGE under reducing conditions and subsequently transferred to nitrocellulose membranes (Protan). Primary antibodies were incubated overnight at 4 °C (Supplemental Table 1). Next, the blots were incubated with a goat-anti-rabbit/mouse IgG Alexa Fluor 680 conjugated secondary antibody (Thermo Fisher Scientific). α-Tubulin was used as loading control. The fluorescent signals were analyzed with the Odyssey CLx Infrared Imaging System (LI-COR Biosciences) and Image Studio Analysis Software Version 4.0.

All experiments, including MTT assays, cell cycle assays and western blots, were performed in duplicate.

### Mutation analysis

For all 4 SyS cell lines the mutation hot spots of *TP53* (exon 4–9) were analyzed by PCR amplification using the AmpliTaq Gold 360 Master Mix (Life Technologies) with 1 μl DNA and the following program: 95 °C (10 min); 95 °C (30 s), 58 °C (30 s), 72 °C (1 min), 38 cycles; and 72 °C for 7 min. Samples were submitted to Sanger DNA sequencing using the BigDye Terminator reaction mix, and samples were analyzed on the 3730 Sequence Analyzer (Applied Biosystems). The known *CTNNB1* G34L mutation was verified in the SYO-1 cell line. All exons (1–5) of *CCND1* were sequenced as described previously.^20^

## Results

### Clinical Data

Only 3 of 43 patients (7 %) were treated with neoadjuvant chemotherapy (including 1 patient <18 years), none had neoadjuvant radiotherapy, and of 14 % treatment status was unknown. Survival data was available for 31 patients (72 %).

### Protein Evaluation by immunohistochemistry and western blot

A total of 43 primary tumors were immunohistochemically stained for cyclin D1, p21, p27, p16, β-catenin, phospho-Rb, and Rb (Fig. 1a, Supplementary Fig. 1). Of the patient samples, 72.1 % had detectable expression of nuclear cyclin D1 (Table 1). Expression of both nuclear phospho-Rb and nuclear β-catenin was an indicator of poor prognosis (Fig. 1c, *p* < 0.05). We observed a significant correlation between Rb and phospho-Rb (*p* = 0.005). No correlation was found between expression of β-catenin and phospho-Rb.

**Table 1:** Patient characteristics

Parameter	Frequency (%) *n* = 43
Age	
Children < 18 years	10 (23.3 %)
Adults ≥ 18 years	33 (76.7 %)
Sex	
Male	28 (65.1 %)
Female	15 (34.9 %)
Tumor localization	
Extremities	27 (62.8 %)
Head/neck	6 (14.0 %)
Other	5 (11.6 %)
Unknown	5 (11.5 %)
Histology	
Monophasic	17 (39.5 %)
Biphasic	13 (30.2 %)
Unknown	13 (30.2 %)
Neoadjuvant treatment	
None	34 (79.1 %)
Chemotherapy	3 (7.0 %)
Radiotherapy	0
Unknown	6 (14.0 %)
Expression of nuclear cyclin D1	
Negative	12 (27.9 %)
Positive	29 (67.4 %)
Missing	2 (4.7 %)
Expression of nuclear Rb	
Negative	33 (76.7 %)
Positive	9 (20.9 %)
Missing	1 (2.3 %)
Expression of nuclear phospho-Rb	
Negative	31 (72.1 %)
Positive	11 (25.6 %)
Missing	1 (2.3 %)
Expression of cytoplasmic p16	
Low	27 (62.8 %)
High	12 (27.9 %)
Missing	4 (9.3 %)
Expression of nuclear p21	
Negative	34 (79.1 %)
Positive	6 (14.0 %)
Missing	3 (7.0 %)
Expression of cytoplasmic p27	
Low	25 (58.1 %)
High	16 (37.2 %)
Missing	2 (4.7 %)
Expression of β-catenin	
Nuclear	
Negative	11 (25.6 %)
Positive	30 (69.8 %)
Missing	2 (4.7 %)
Cytoplasmic	
Negative	7 (16.3 %)
Positive	34 (79.1 %)
Missing	2 (4.7 %)
Membranous	
Negative	30 (69.8 %)
Positive	11 (25.6 %)
Missing	2 (4.7 %)

The 4 SyS cell lines were evaluated for expression of the same proteins by western blot. Expression levels differed between the cell lines (Fig. 1b). The Yamato-SS had the highest expression of cyclin D1, whereas the CME-1 cell line expressed a high level of p16, with a corresponding lower level of cyclin D1.

### Genetic Alterations

**Fig. 2:**
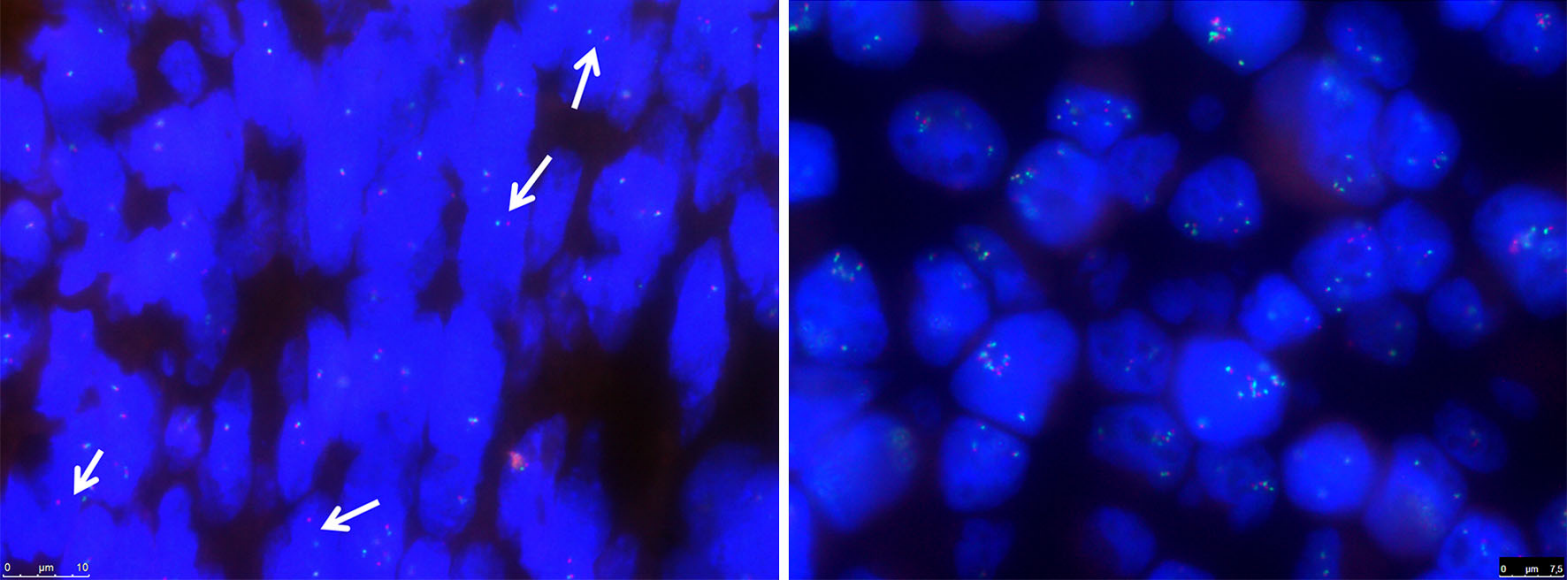
Genetic alterations. *CCND1* breakage in patient tumor sample (*left*), as indicated by the arrows and *CCND1* amplification in the Yamato-SS cell line (*right*) by FISH analysis

FISH analysis showed sporadic breakage, in 10–20 % of the cells, of *CCND1* in 15 % of the patient samples. Additionally, an 8-fold amplification of *CCND1* was found in the Yamato-SS cell line (Fig. 2). The known *CTNNB1* G34L mutation was confirmed in the SYO-1 cell line.^14^ The Yamato-SS cell line harbored a homozygous *TP53* mutation in exon 8 (c.817C > T/p.R273C, rs121913343). This deactivating *TP53* mutation could explain the lack of downstream target p21 found by western blot. None of the cell lines harbored a mutation in *CCND1*.

### Palbociclib sensitivity in vitro

**Fig. 3:**
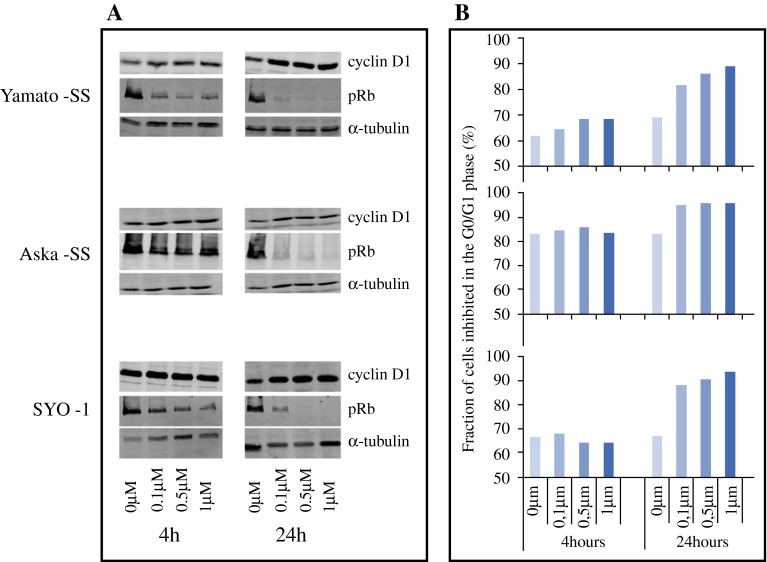
Western blot and cell cycle analysis. **A** Protein evaluation by western blot shows a decrease in phospho-Rb at increasing concentrations of palbociclib at different time points in the Yamato-SS, Aska-SS and SYO-1 cell lines. As expected, no effect can be seen on cyclin D1 expression. **B** This *column* shows the cell cycle analysis. The *bars* indicate the G0/G1 phase, and show an increase in G0/G1 arrest in Yamato-SS, Aska-SS and SYO-1 cell lines

MTT assays revealed a dose-dependent growth inhibition by palbociclib in all cell lines, in which 3 cell lines (Yamato-SS, Aska-SS, and SYO-1) had an IC_50_ value of 0.83 µM ± 0.16, 0.98 µM ± 0.17, and 0.12 µM ± 0.01, respectively. Since a concentration below 1.2 µM is considered feasible as plasma concentration in patients, IC_50_ values below this concentration are considered sensitive.^23^ The CME-1 cell line had an IC_50_ value of 8.0 µM ± 1.32 and is therefore regarded resistant to palbociclib. Palbociclib resulted in a concentration-dependent reduction of phospho-Rb levels, already observed after 4 h of incubation. Inhibition of Rb phosphorylation was best seen at 24 h (Fig. 3a). Evaluation of the G1 arrest was done by analyzing DNA content of the cells (propidium iodide staining) (Fig. 3a). A G0/G1 arrest was induced in the 3 sensitive cell lines at 24 h. In the resistant CME-1 cell line no effect of palbociclib on the cell cycle was observed (data not shown).

## Discussion

**Fig. 4:**
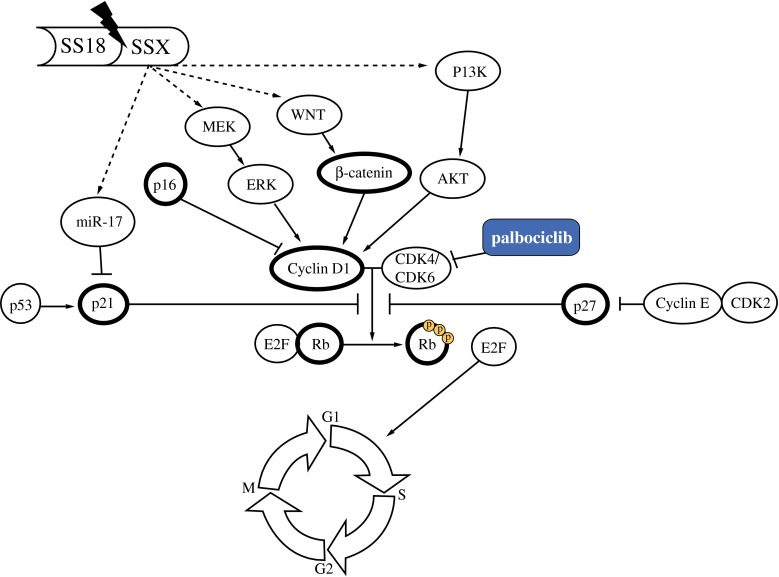
Cyclin D1-CDK4/6-Rb axis. *Highlighted* are the proteins that have been investigated by immunohistochemistry and western blot

Palbociclib is a well-tolerated drug with significant progression-free survival (PFS) benefit in patients, in combination with letrozole, for the treatment of postmenopausal women with ER-positive, HER2-negative advanced breast cancer as initial therapy for their metastatic disease.^24^ Patients with STS have been tested for palbociclib as well. A phase II study in well-differentiated and dedifferentiated liposarcoma patients showed a partial response in 1 of 29 evaluable patients, with a median PFS of 18 weeks.^22^ Even though the cyclin D1-CDK4/6-Rb axis has been thought to play a role in SyS, so far no studies have reported on the effects of inhibiting this axis in SyS. Here, we used patient tumor samples and 4 SyS cell lines to evaluate genetic alterations of *CCND1* and the expression patterns of the proteins playing a possible role in deregulation of this axis (Fig. 4). We showed *CCND1* amplification in a SyS cell line and *CCND1* rearrangement in a small percentage of cells in a subset of patient samples, although possible fusion partners and the significance of these rearrangements in a small subset of tumor cells remain elusive. Even though cyclin D1 is regularly reported to be a downstream target of several possibly activated pathways in SyS, potentially induced by its fusion gene, the exact working mechanism of the translocation in respect to cyclin D1 remains unknown. We show a significant relation between nuclear β-catenin expression and overall survival of SyS patients, which is in line with previous studies.^16^,^25^ We provide information on the different localizations of β-catenin expression (Table 1); however, as only the nuclear expression has effect on cyclin D1, we used this localization for prognostic evaluation. In addition, we show that nuclear expression of phospho-Rb correlates with overall survival. This might be a new potential prognostic indicator, and further validation is needed in a larger cohort.

Based on the presumed involvement of deregulated cyclin D1 activity in SyS, we used palbociclib, a potent inhibitor of this axis, in 4 SyS cell lines. Interestingly, previous studies showed that palbociclib was considerably less active in a number of non-synovial soft tissue sarcoma cell lines (IC_50_ values of 8.95–26.63 µM), suggesting that an aberrant cyclin D1-CDK4/6-Rb axis is a specific feature for the SyS subtype of soft tissue sarcomas.^26^ One SyS cell line had a noticeably higher IC_50_ (CME-1), probably explained by the immortalization with SV40 large T antigen. This binds to p53 and affects p53 transcriptional activity. SV40 large T antigen also affects Rb activity, which leads to a bypassing of the G1/S checkpoint with a resulting constitutive activation of the cell cycle.^27^,^28^

As palbociclib does not result in a 100 % G1 arrest, combination therapy will be necessary to induce the effect, which may thus potentially translate into higher activity in the clinic. Numerous potential combination therapies can be investigated, including various chemotherapeutics, small molecule inhibitors, and targeted antibodies, etc. Very recently, a new insight in the translocation and the Wnt pathway was established with a study reporting that the translocation induces a partial Wnt expression signature in the absence of Wnt ligands, using β-catenin as a transcriptional de-repressor.^29^ With these functional studies, insight into the pathophysiology improves, which will hopefully result in rationale-based combination treatments.

In conclusion, this study shows an extensive overview on the activity of the cyclin D1-CDK4/6-Rb axis in SyS, in which genetic deregulation of *CCND1* is rare. Further research is necessary to examine the potential signaling mechanisms between this axis and β-catenin, as this was not subject of our research. Nuclear phospho-Rb and β-catenin expression is correlated negatively with overall survival in SyS patients, which has a potential prognostic role, but should be validated in a separate dataset. Palbociclib induces a cell cycle arrest with corresponding phospho-Rb decrease in SyS cell lines, making palbociclib a potential treatment option for SyS patients. Whether this effect can be enhanced by combination treatments deserves further preclinical investigations.

## Electronic supplementary material

Below is the link to the electronic supplementary material.

Supplementary figure 1: Immunohistochemical scoring system. Nuclear staining of the proteins cyclin D1, Rb and p21 was scored as negative or positive. The cut off was set at staining in at least 20% of the cells; if less than 20% of the cells were stained, the sample was considered negative. Nuclear and cytoplasmic staining for p16 and p27, respectively, was scored as negative/mild (low), or moderate/strong (high) in at least 20% of cells. All images are taken at x200 magnification. Supplementary material 1 (TIFF 9971 kb)

Supplementary material 2 (DOCX 13 kb)
