# Supplementary figures and images for: Targeting Cyclin-Dependent Kinases in Synovial Sarcoma: Palbociclib as a Potential Treatment for Synovial Sarcoma Patients
Source: Ann Surg Oncol. 2016 Jun 22;23:2745–52. doi: 10.1245/s10434-016-5341-x (PMC4972869; doi:10.1245/s10434-016-5341-x)

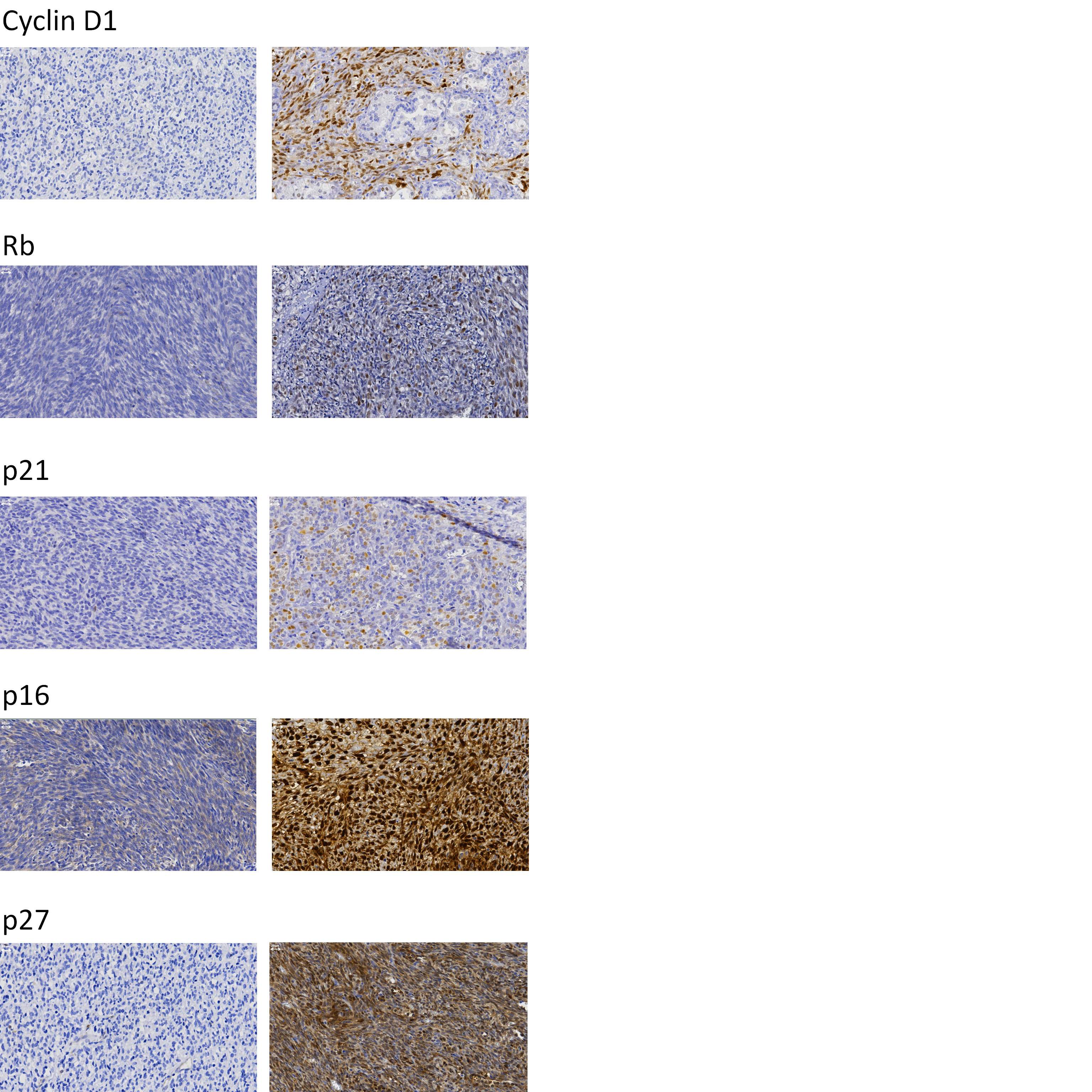

Supplement: Supplementary file 1 — Supplementary figure 1: Immunohistochemical scoring system. Nuclear staining of the proteins cyclin D1, Rb and p21 was scored as negative or positive. The cut off was set at staining in at least 20% of the cells; if less than 20% of the cells were stained, the sample was considered negative. Nuclear and cytoplasmic staining for p16 and p27, respectively, was scored as negative/mild (low), or moderate/strong (high) in at least 20% of cells. All images are taken at x200 magnification. Supplementary material 1 (TIFF 9971 kb) [file 10434_2016_5341_MOESM1_ESM.tif]
