# Supplementary material for: Targeting Cyclin-Dependent Kinases in Synovial Sarcoma: Palbociclib as a Potential Treatment for Synovial Sarcoma Patients
Source: Ann Surg Oncol. 2016 Jun 22;23:2745–52. doi: 10.1245/s10434-016-5341-x (PMC4972869; doi:10.1245/s10434-016-5341-x)
Supplement: Supplementary file 2 — Supplementary material 2 (DOCX 13 kb) [file 10434_2016_5341_MOESM2_ESM.docx]

Supplementary table 1: antibodies

| \|  \|  \| **Immunohistochemistry** \| \| **Western blot** \| \| --- \| --- \| --- \| --- \| --- \| \| **Antigen** \| **Antibody** \| **Heat Induced Epitope Retrieval (HIER)** \| **Dilution** \| **Dilution** \| \| p21 \| #ms-230-P1, clone DCS-60.2, Thermo Scientific \| 30 min in 10 mM sodium citrate buffer pH6,7 \| 1/20 \| - \| \| p21 \| #2946, clone DCS60,  Cell Signaling Technology \| - \| - \| 1/2000 \| \| p16 \| #550834, clone G175-405, BD Biosciences \| 10 min in 10 mM sodium citrate buffer pH6 \| 1/4 \| 1/300 \| \| β-catenin \| #610153, clone 14/Beta-Catenin, BD Biosciences \| 10 min in EDTA buffer pH9 \| 1/400 \| 1/2000 \| \| pRb \| #8516, clone D20B12, Cell Signaling Technology \| 10 min in 10 mM sodium citrate buffer pH6 \| 1/200 \| 1/1000 \| \| Rb \| #9309, clone 4H1,  Cell Signaling Technology \| 10 min in 10 mM sodium citrate buffer pH6 \| 1/200 \| - \| \| p27 \| #ab32034, clone Y236, Abcam \| 10 min in 10 mM sodium citrate buffer pH6 \| 1/200 \| 1/1000 \| \| cyclin D1 \| ILM 30442 C1, clone SP4, Immunologic \| 30 min in 10 mM sodium citrate buffer pH6,7 \| 1/20 \| 1/300 \| |
| --- | --- | --- | --- | --- | --- | --- | --- | --- | --- | --- | --- | --- | --- | --- | --- | --- | --- | --- | --- | --- | --- | --- | --- | --- | --- | --- | --- | --- | --- | --- | --- | --- | --- | --- | --- | --- | --- | --- | --- | --- | --- | --- | --- | --- | --- | --- | --- | --- | --- | --- |
